# The Drink Question in France

**Published:** 1890-02

**Authors:** 


### The Drink Question in France.

The recent Anti-Alcohol Congress in Paris showed that the dram shops of Paris have risen since 1880 from 24,000 to 29,900. In thirty years the consumption of alcohol has been trebled, and as much as 36,000,000 gallons have been manufactured out of potatoes for the French market. This shows an average yearly consumption of over twelve quarts per adult man. The consumption of alcohol doubled between 1875 and 1885. The congress voted a resolution that inebriates should be treated as mad, and that prison hospitals should be created for them. It was also resolved that the governments of the world should be asked to impose a prohibitive duty on alcohol, and exempt from duty tea, coffee, and other ingredients for temperance drinks.

